# respiTrack: Patient-specific real-time respiratory tumor motion prediction using magnetic tracking

**DOI:** 10.1007/s11548-020-02174-3

**Published:** 2020-04-28

**Authors:** Yusuf Özbek, Zoltán Bárdosi, Wolfgang Freysinger

**Affiliations:** Medical University of Innsbruck, Innsbruck, Austria

**Keywords:** Real-time tumor tracking, Respiratory motion, Prediction optimization, Magnetic tracking

## Abstract

****Purpose**:**

An intraoperative real-time respiratory tumor motion prediction system with magnetic tracking technology is presented. Based on respiratory movements in different body regions, it provides patient and single/multiple tumor-specific prediction that facilitates the guiding of treatments.

****Methods**:**

A custom-built phantom patient model replicates the respiratory cycles similar to a human body, while the custom-built sensor holder concept is applied on the patient’s surface to find optimum sensor number and their best possible placement locations to use in real-time surgical navigation and motion prediction of internal tumors. Automatic marker localization applied to patient’s 4D-CT data, feature selection and Gaussian process regression algorithms enable off-line prediction in the preoperative phase to increase the accuracy of real-time prediction.

****Results**:**

Two evaluation methods with three different registration patterns (at fully/half inhaled and fully exhaled positions) were used quantitatively at all internal target positions in phantom: The statical method evaluates the accuracy by stopping simulated breathing and dynamic with continued breathing patterns. The overall root mean square error (RMS) for both methods was between $0.32\pm 0.06~\hbox {mm}$ and $3.71\pm 0.79~\hbox {mm}$. The overall registration RMS error was $0.6\pm 0.4~\hbox {mm}$. The best prediction errors were observed by registrations at half inhaled positions with minimum $0.27\pm 0.02~\hbox {mm}$, maximum $2.90\pm 0.72~\hbox {mm}$. The resulting accuracy satisfies most radiotherapy treatments or surgeries, e.g., for lung, liver, prostate and spine.

****Conclusion**:**

The built system is proposed to predict respiratory motions of internal structures in the body while the patient is breathing freely during treatment. The custom-built sensor holders are compatible with magnetic tracking. Our presented approach reduces known technological and human limitations of commonly used methods for physicians and patients.

## Introduction

The advantages of surgical tracking technology are used for real-time tumor or organ motion prediction aiming to guide the surgeries or therapies with minimal damage to the surrounding tissue around the target. In particular, treatments in stereotactic ablative radiotherapy (SABR) or stereotactic body radiation therapy (SBRT) while the patient is breathing freely are an important concern in clinical workflows for the safe and effective provision of precision radiotherapy, computer-assisted tumor surgery and biopsy interventions [1–4]. Existing approaches that are requiring extensive training for the patients and physicians such as respiratory gating and breath hold are often a constraint. For abdominal compression, used pneumatic belts or mechanical pressure systems are inconvenient for the patients when considered the long therapy sessions and periods.

**Fig. 1:**
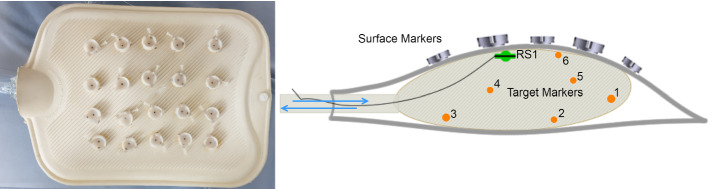
Left: Respiratory model with the fixed SHs. Right: Inside of the model. To place the markers in the balloon, the balloon is turned inside–out and 6 X-Spot CT skin markers (${\oslash }$ 1.5 mm, lead-free metallic pellet), 1 titanium Rhinospider ball (${\oslash }$ 4 mm) isocentrically carrying a cylindrical 5D magnetic sensor (${\oslash }$ 0.5 mm, L 8 mm), are glued on the inner wall of the balloon (maximum inflation ${\oslash }$ 120 cm). Thereafter the balloon is turned outside–in. For inflating with a water blaster ($82\times 5\times 15$ cm), a flexible silicone tube (${\oslash }$ 20 mm, L 200 cm) is connected to the balloon

We present a patient-specific approach that predicts internal tumor motions using real-time tracked skin sensors with magnetic tracking while giving to patient relaxed freely breathing condition. The respiratory cycle of the patient and thus the 3D temporospatial movements of the internal targets are observed from patient’s 4D-CT preoperatively. Our optimization technique [5] and custom-made surface sensor holder (SH) allow to determine the best possible localization and number of sensors to be placed on the patient’s surface to predict single or multiple tumor movements at the best possible locations. Accurate positioning of the sensors at the proposed SHs preoperatively allows submillimetric registration accuracy and thus clinical acceptable real-time prediction in intraoperative phase.

## Methods

This section describes the hardware and software components of the respiratory motion prediction system, possible clinical workflow and the implementation of respiTrack.

## Components

### Custom respiratory system model

**Fig. 2:**
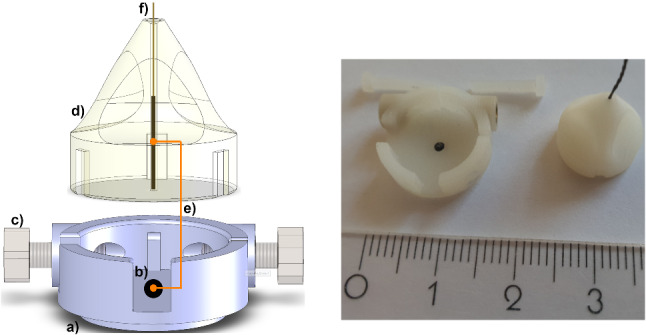
Left: (**a**) Main part of SH. (**b**) A X-Spot CT marker placed in the main part concentrically. (**c**) Two M2 screws are applied from both directions to establish a rigid setup once an EM-SH is placed in the main SH. (**d**) The EM-SH holds the tracking sensor perpendicular to CT marker and plugged into the main part, so that the offset (**e**) between sensor and CT marker center is determined. (**f**) 5D tracking sensor is fixed in the EM-SH concentrically. Right: SH with an EM-SH and tracking sensor shown in cm. The parts (**a**) and (**b**) are intended to use for off-line prediction after image acquisition and all together in intraoperative real-time prediction. The SHs are distributed homogeneously on the surface with $\approx 3~\hbox {cm}$ distance

To replicate the human respiratory system artificially and to predict the tumor motion based on the respiratory simulation, a custom-made realistic phantom model was built (Fig. 1). The standard rubber hot-water bottle simulates, e.g., abdominal region of the human body that contains a spherical rubber balloon inside to simulate a moving organ. The respiratory cycle of the model is performed by inflating/deflating the balloon using water blaster manually. A silicone tube connects the balloon to water blaster. The SHs (Fig. 2) are fixed on the model surface (surface fiducials/markers). The balloon, inside the bottle, contains different markers in size internally (target markers) distributed in various locations and replicates moving tumors along different movement directions such as vertical, lateral or longitudinal.

### Sensor holder

Several real-time movement prediction techniques apply external surface sensors [6–8]. However, those sensors are placed at discretionary locations and distributed empirically using a fixed number of sensors for each patient that may be sub-optimal for the real-time prediction accuracy [9].

The concept of the custom-made SH can improve the real-time prediction accuracy in intraoperative phase by optimizing the spatiotemporal distribution and alternating number of surface markers to use for each patient and multiple tumor movement prediction with respect to their predictive power in the preoperative phase. The improved SH design enables switching the tracking sensors while maintaining the same sensor origin and provides off-line prediction preoperatively using surface fiducials in SH and the pre-trained predictors with magnetic tracking during the intervention after the known relative transformation between the surface fiducials and the inserted real-time tracker sensor.

The main component of the SH consists of a X-Spot CT skin fiducial (Beekley Medical, Bristol-CT), centered in a sensor attachment point. During the preoperative phase, $\approx$10–25 of these main SHs are fixed on the patient’s surface at randomized candidate locations according to the interested body region. In the intraoperative phase, the main SH holds an magnetic sensor holder (EM-SH) within a tracking sensor (NDI Aurora, 40 Hz measurement rate, Northern Digital Inc., Canada) concentrically. The SHs provide user error-free rigid body image-to-patient registration [10], and therefore, more accurate real-time motion prediction can be achieved. A 6D sensor was used as a dynamic reference frame (DRF) for the registration. The SH design allows both automated localization in 4D-CT patient images and during real-time motion tracking. A fully automated registration process eliminates possible user errors and allows high-accuracy registrations potentially with submillimetric errors on the target.

### Rhinospider

**Fig. 3:**
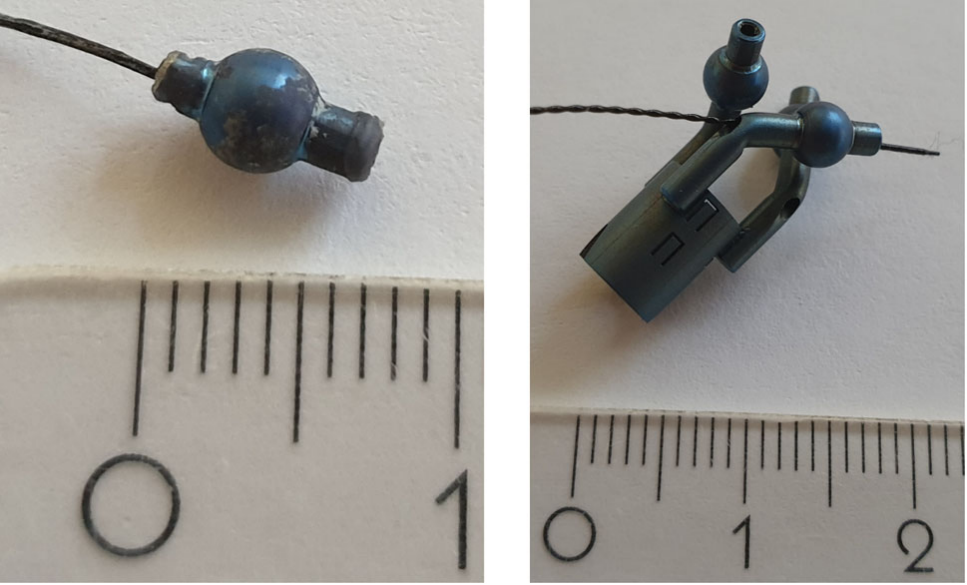
Left: RS ball within sensor placed in the phantom model. Right: Original RS device design, inserted in the posterior nasal cavity preoperatively. The sensor tip is shifted along the ball for better visibility. Both shown in cm

Rhinospider (RS) is a novel registration technique used in combination with magnetic tracking to determine the accurate fiducial localization and optimize the workflow for patient-to-image registration [11]. In this work, a RS ball was used for the validation of the real-time prediction to determine the correctness of prediction accuracy and identify the positional deviations between the tumor (predicted RS ball center) and the center of tracked 5D sensor in the ball. In the RS ball, a 5D sensor was attached (both centroids of the RS ball and sensor are matching) (Fig. 1, right and Fig. 3, right) and placed inside of the phantom model before 4D-CT scan (Fig. 3 left). The RS ball was detected and localized in CT image space automatically same as other CT skin/internal markers in the model.

### respiTrack software

A plugin-based prototype software system (respiTrack) featuring preoperative planning (off-line prediction), intraoperative registration, surgical navigation and real-time prediction was developed. All the required modules [12] were implemented using open-source libraries [13–18].

## Workflow

**Fig. 4:**
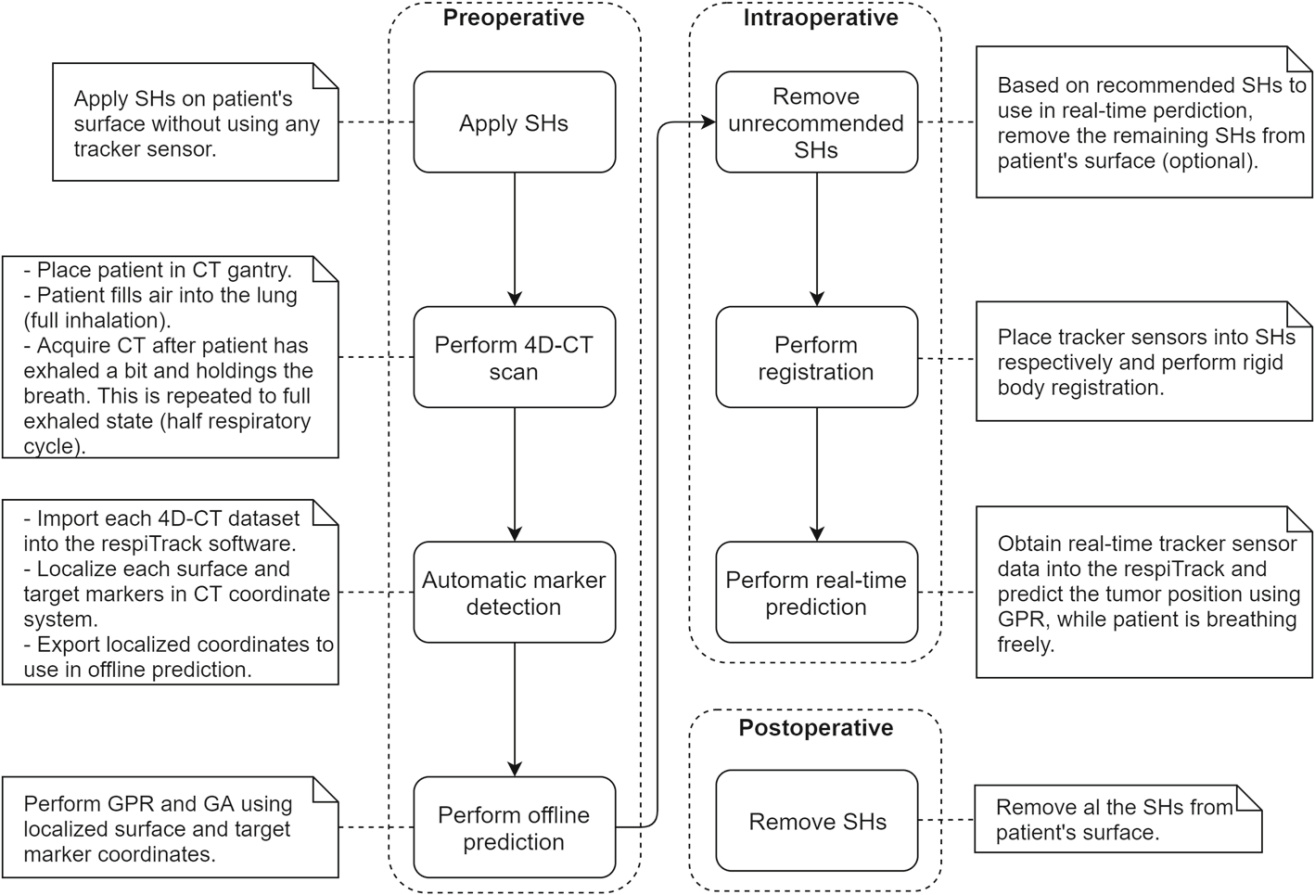
respiTrack workflow

The individual steps (Fig. 4) in respiTrack describe the performed procedures from preoperative until postoperative phase consecutively.

### Data acquisition

For the 4D-CT, a scanner (cardiac scan with SOMATOM Definition Flash; temporal resolution 75 ms; scan time 0.6 s; Siemens healthineers, Austria) at the University Clinic for Radiology in Medical University of Innsbruck was used.

**Fig. 5:**
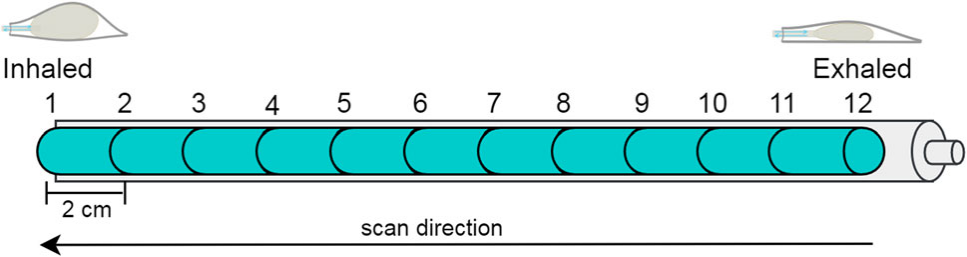
Visualization of the handle

The phantom model with 20 SHs within 7 targets was placed into the CT gantry and held at fully inhaled position by adjusting air in the balloon. (position 1 in Fig. 5). In total, $\approx$10–15 CT scans with discrete time steps of a half breathing cycle were acquired. Before each scan, the air in the balloon was decreased by moving the handle of the water blaster to the next marked position until fully exhaled position was reached. The distance between each marked positions on the handle is 2 cm.

**Fig. 6:**
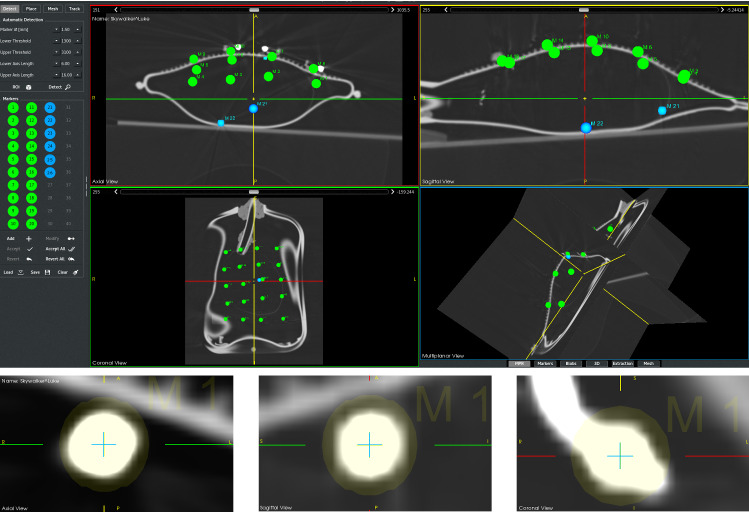
Top: 3D positions of the surface and target marker centroids were detected and localized automatically and managed in a fiducial list for each 4D-CT phase, respectively. 20 green spheres on the images show the localized and accepted surface markers and blues, candidate target markers to be accepted. Bottom: Enlarged CT views of detected RS ball. It is clearly visible that the crosshair (indicates the localization position) is located in the center of the ball

The slice thickness for each CT image ($512\times 512$ px) was 1.0 mm, and the 12 discrete CT phases consist of 303 images with $0.488\times 0.488\times 0.488$ mm pixel spacing. The 4D-CT scan was loaded into the respiTrack software and visualized as standard DICOM view (axial, sagittal, coronal and multiplanar) (Fig. 6).

### Marker detection and localization

The automatic localization of the surface and target markers was performed using a GPU accelerated volumetric detection method [19] that uses morphological opening and closing operators.

To determine the marker centroids, each 4D-CT image set was loaded into the respiTrack and thresholded with given Hounsfield unit parameter that binarizes the images. A virtual structuring sphere element with given physical dimensions and appropriate scale given the voxel size of the image, was generated and applied to the images. A geometry filter selects best candidates based on the shape and size on the determined spherical blobs and calculates the 3D positional centroids in CT image space.

The detected marker locations for each 4D-CT phase were exported (input and target data) and used for training data during prediction. The observations represent the respiratory cycle of the patient and the 3D temporospatial movement variance of all surface and target markers for a half breathing cycle in 12 discrete time steps (Table 1). The most marker movement amplitude was observed in *z* image plane (SI-superior/inferior), while less movements were observed in *y* (AP-anterior/posterior) and *x* planes (LR-left/right), respectively. The total movements assure consequently that internal marker movements are replicating very similar respiratory organ motions with internal organs of a human body, such as heart, lung, liver, trachea, prostate and spine [20–23]. The temporospatial movements of surface markers behave similar to target marker movements. Maximum movement in the AP plane was observed for Marker 9 $(-18.25\;\hbox {mm})$ and minimum for marker 15 $(1.20\;\hbox {mm})$. In SI plane, maximum was observed for marker 6 $(1.01\;\hbox {mm})$ and minimum for 13 $(0.02\;\hbox {mm})$, while in LR plane, maximum movement for marker 10 $(2.83\;\hbox {mm})$ and minimum for 20 $(0.26\;\hbox {mm})$.

**Table 1:** 3D temporospatial movement variations of the internal target markers (centroids) during 4D-CT imaging in mm

Target	1	2	3	4	5	6	RS1
LR	4.00	$-$ 1.91	1.00	0.14	0.51	0.29	7.75
AP	$-$ 3.67	1.71	0.10	$-$ 3.65	$-$ 11.25	$-$ 6.67	$-$9.56
SI	11.04	$-$ 4.39	$-$ 0.18	0.01	0.54	9.82	11.35

### Respiratory motion prediction and optimization

On the basis of known spatial coordinates of surface and target markers in 4D-CT image space, the optimal number of sensors to be used for desired single or multiple tumors and their best possible sensor locations to be placed on the patient’s surface were determined in off-line prediction phase. This optimization process eliminates one of the major error sources for the prediction accuracy as configurable for each tumor and patient individually. During real-time prediction, the 5D sensors were applied in the corresponding SHs recommended by off-line prediction and tracked while patient is breathing freely.

### Off-line prediction

The exported spatial coordinates of both marker locations in 4D-CT reference frame (*k* time-series, each with *T* time steps and 3D output dimensions in *x*, *y*, *z* yields the time-series $p \in R^{T \times 3}$) were used to determine the optimal surface sensor locations preoperatively by using multi-objective genetic algorithm (GA)-based feature selection method [24, 25], which trains an accurate prediction of tumor motion from few optimally positioned SHs.

An individual *I* in total population (possible solution in metaheuristic search) during the GA search is represented by a chromosome of a *k*-dimensional binary vector $I = \lbrace 0, 1 \rbrace ^{k}$, where the *n*th bit (gene) in chromosome represents whether the *n*th SH marker is used for prediction 1 or not 0.

If a SH marker is selected to use, its 3D positional coordinates within the CT reference frame are added to the input coordinate set used for prediction. This yields $3 \times M$-dimensional input feature for each time step, where *M* is the number of enabled markers within the individual. For each *I*, the fitness function is defined by multi-objective function $F(I) = \left( F_1(I), S(I) \right)$. The primary component is given by the weighted sum:

$$\begin{aligned} F_1(I) = E(I) + \alpha * \min (0, S(I) - K), \end{aligned}$$

where *E*(*I*) is the average RMS error between the predicted and target locations using *X* as the input feature set over a threefold cross-validation on the *T* time steps, *S*(*I*) is the number of features enabled, *K* is the maximum preferred number of enabled surface markers, and $\alpha$ is a scaling parameter, which balances the trade-off between additional prediction error and the number of enabled marker. This setup leads to an optimization goal of finding the minimum achievable prediction error with as few marker as possible, but softly punishing configurations that have more than *K* enabled sensors. The GA in respiTrack was configured with generation size 60 (termination criteria), population size 600, crossover proba 0.5, mutation proba 0.2, cv independent proba 0.5 and mu independent proba 0.05.

For each *I*, the predictions were evaluated using 3 Gaussian process regressors (GPR) ($G_i: X \rightarrow t_i$, $i = {1,2,3}$) for each coordinate of the target *y*, with kernel $C * \hbox {SE} + W$ where *C* is constant kernel $\sigma ^{2}$, *SE* is squared exponential $\sigma ^2 \exp \left( -\frac{(x,x')^2}{2l^2}\right)$, and *W* is white noise kernel $\sigma ^{2}l_{n}$ [26].

The *C* kernel was configured with variance 1.0 and bounds $(1e-3,1e3)$, while *SE* kernel with length scale 10.0, bounds $(1e-2, 1e2)$ and *W* kernel with noise variance 0.1 and bounds $(1e-10, 1e+0.5)$. The GPR was configured with normalized target-data mean value without an optimizer. Off-line prediction was repeated ten times for each individual target *y*, respectively, that gave same recommendation SH list after each run.

### Real-time prediction

The intraoperative image-to-patient registration was established, while 50 sensor location readings (relative to DRF) were averaged for every attached single sensor (dependent on the number of recommended sensor list *S*(*I*) for an individual target *y*) and patient maintains a fixed position relative to the field generator with or without breath held. The combined sensors and SH marker coordinates were matched $T_t,_p$ to find the minimum registration error (FRE) [27].

**Fig. 7:**
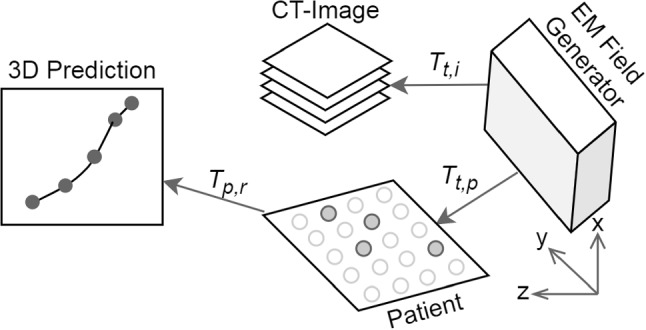
Built transformation chain in respiTrack

During real-time prediction, each observed sensor readings $L_i \in S(I)$ were transformed from tracker to image coordinate system proposed to use as test data $T_p,_r$ in GPR by $(\overrightarrow{V}_{L_i(x,y,z,1)})^T*R$, where $\overrightarrow{V}$ was a $1\times 4$ vector for each individual sensor coordinate in tracker coordinate space and *R* is a $4\times 4$ matrix observed through rigid-body registration (Fig. 7). The GPR was applied with the same kernel and input-data $L \in X$ to a desired target *y* with read real-time test data $L_i$.

## Evaluation

### Experimental setup

For each target, the recommended number of SHs and their identified locations were used, respectively. The patient was then positioned in the FOV of tracker, and real-time sensor data (test data) were observed. The prediction accuracy was validated using RS ball within located sensor, which was intended to use as a tracked target marker (Target RS1 in all tables).

### Evaluation procedure

**Fig. 8:**
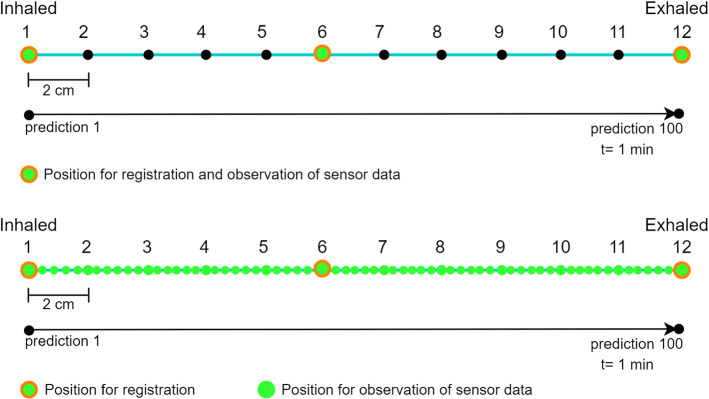
Visualizations of statical (top) and dynamical (bottom) evaluation patterns

Two different validation methods were applied. In statical method, the prediction for a selected target was determined in different three fixed positions by reading test data without simulating any breathing. For this, the following steps were executed:

1. Load first patient dataset into respiTrack (4D-CT scan in fully inhaled position) (Fig. 8, top).
2. Fill air in the model until marked position on the handle regarding number of loaded dataset and perform patient-to-image registration.
3. Perform real-time prediction for all targets, respectively, while holding the handle on the fixed position without changing the air in the model.
4. Repeat same experiment for half inhaled (6th) and fully exhaled (12th) dataset.

In dynamical method, the prediction was determined with the same steps (except 3) above while changing the amount of air in the patient between handle positions 1 and 12 repetitively (Fig. 8, bottom). The operator was synchronizing his/her relaxed breathing cycle while simulating the inhalation and exhalation with the patient. Each validation procedure was repeated five times, and standard deviation (SD) for each run was calculated. The correctness of prediction accuracy was determined for target RS1 while comparing the predicted and real-time sensor reading positions (Table 6). For each validation step, 100 predictions were accomplished that took $\approx$ 1 min. Each individual prediction took 0.62 s. The registrations were established on the three different marked positions, which were not showing any significant influence on registration accuracy but on prediction accuracy (See prediction RMS columns “Reg. at 6th pos.” in Tables 3 and 5). Test data were observed during simulated breathing.

## Results

Various external surface markers were discovered to predict temporospatial movements of 7 internal targets from best possible SH locations. The resulting number of SHs was decided by the feature selection algorithm from 20 SHs in total, distributed on the patient’s surface, and predictions were performed by the heuristic GPR algorithm. The best prediction accuracy was observed by combined kernels with their generalization properties. Tables 2 and 3 represent the resulting off-line and real-time prediction accuracy for each target in the phantom. Each input marker in the recommended SH list was processed with an individual target, respectively.

Leave-one-out cross-validation procedure (LOO) [28] was applied to validate off-line prediction from both input and target marker positions $Nx(L*D)$, respectively, where *N* is the total number of 4D-CT phases, *L* is the total number of recommended SHs, and *D* is the dimension of the data. The training of the predictor was performed on $N-1xD$th of the *NxD* input data, and the prediction was tested on remaining *N*th test data for an individual target *y*. This process was repeated *N* times each time leaving out a different pair to use as the single test case.

Instead of recommended SHs, randomly selected SH locations were used to cross-validate the results and to investigate different location of SHs on the accuracy effect between the motions of SHs and a target under various registration patterns (Tables 4 and 5).

**Table 2:** Off-line prediction results associated with proposed LOO

Target	Optimal sensor number	Recommended SHs	Kernel	Data	LOO Mean RMS
1	4	1–7–15–16	$C*\hbox {SE}+W$	$Nx(L*D)$	0.085
2	6	7–12–16–17–19–20			0.146
3	5	6–8–12–14–16			0.072
4	4	4–8–17–18			0.067
5	4	1–2–5–13			0.093
6	5	3–6–8–16–18			0.41
RS1	3	8–11–12			0.207

The RMSs in mm show the deviations in each time step when using the recommended SH list

**Table 3:** Real-time prediction results using recommended SHs in mm

Target	Recommended SHs	Pred. size	Data	Mean Reg. RMS	Statical validation			Dynamical validation			
					Reg. at 1st pos.	Reg. at 6th pos.	Reg. at 12th pos.	Reg. at 1st pos.	Reg. at 6th pos.	Reg. at 12’th pos.	
					Pred. RMS	Pred. RMS	Pred. RMS	Pred. RMS	Pred. RMS	Pred. RMS	Mean RMS
1	1–7–15–16	3000	Lx(R*D)	$0.6\pm 04$	3.95	2.18	4.06	3.99	3.63	4.45	$3.71\pm 0.79$
2	7–12–16–17–19–20				1.17	1.01	2.08	1.28	1.24	1.34	$1.35\pm 0.37$
3	6–8–12–14–16				0.31	0.25	0.44	0.31	0.30	0.33	$0.32\pm 0.06$
4	4–8–17–18				0.96	0.67	1.36	0.94	0.87	1.05	$0.97\pm 0.22$
5	1–2–5–13				3.16	1.98	3.79	2.88	2.86	3.47	$3.02\pm 0.62$
6	3–6–8–16–18				1.97	1.57	3.52	2.21	2.05	1.87	$2.16\pm 0.68$
RS1	8–11–12				3.56	2.09	4.19	3.53	3.19	3.25	$3.30\pm 0.69$

**Table 4:** Off-line prediction results with randomly selected SH/s than recommended by GA

Target	Optimal sensor number	Random SHs	Kernel	Data	LOO Mean RMS
1	4	2–5–10–12	$C*\hbox {SE}+W$	$Nx(L*D)$	0.157
2	6	1–3–5–11–12–15			0.154
3	5	2–3–7–16–20			0.080
4	4	3–7–15–18			0.72
5	4	3–7–15–18			0.106
6	5	1–3–4–17–20			0.174
RS1	3	8–13–17			0.235

The results are clearly showing that recommended SHs locations provide better accuracy

**Table 5:** Real-time prediction results using randomly selected SHs in mm. Dynamical validation was not necessary since the statical validation results already show better prediction accuracy (Table 3)

Target	Random SHs	Pred. size	Data	Mean Reg. RMS	Statical validation			
					Reg. at 1st pos.	Reg. at 6th pos.	Reg. at 12’th pos.	
					Pred. RMS	Pred. RMS	Pred. RMS	Mean RMS
1	2–5–10–12	3000	$Lx(R*D)$	$0.6\pm 0.4$	4.16	2.17	3.79	$3.37\pm 1.12$
2	1–3–5–11–12–15				1.19	1.01	2.10	$1.43\pm 0.34$
3	2–3–7–16–20				4.31	3.47	3.76	$3.84\pm 0.18$
4	3–7–15–18				1.18	0.69	1.41	$1.09\pm 0.13$
5	3–7–15–18				3.21	1.98	3.94	$3.04\pm 0.98$
6	1–3–4–17–20				4.03	2.73	5.85	$4.20\pm 2.45$
RS1	8–13–17				3.84	2.11	5.21	$3.72\pm 2.41$

**Fig. 9:**
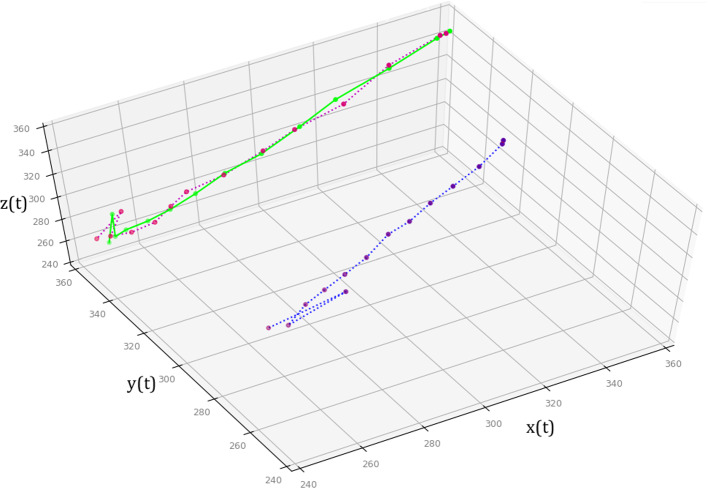
Visualization of the prediction for target 1. Green line indicates prediction, red dotted line: measured positions of target marker in the first 4D-CT phase, blue dotted line: movement of the recommended marker group (1–7–15–16) at 14 different time steps

## Discussion and conclusions

In this paper, we proposed a real-time respiratory motion prediction system that uses surface sensors to predict internal tumor motions (Fig. 9). For magnetic tracking, provided nondisposable SH concept ensures user error-free registration and uninterrupted data flow without line-of-side limitations. Automatically identifying best possible sensor locations on the patient’s surface preoperatively that shows the distribution of recommended sensor locations having a high correlation between the surface motion and the internal tumor motion, provides better target accuracy using less numbers of external sensors to use, e.g., in the thorax or abdominal regions in intraoperative phase. In particular, enabling free breathing for the patient during treatments and multiple tumor prediction without additional workload on the medical staff, enhance common workflows in such treatments.

**Table 6:** Measured 3D deviations between predicted and read sensor data for target RS1 in Table 3 with overall $\pm 0.05~\hbox {mm}$ SD that occurs due to time-variant expansion of the balloon

Target RS1	Pred. size	Variance
LR		0.29
AP	500	0.17
SI		0.29

Our internal tests with the system serve reliable prediction accuracy and show a promising potential to be used in SABR and SBRT treatments or tumor and biopsy surgeries. The system overcomes many of the limitations of common clinical approaches and can be integrated into the existing clinical workflows in the medical environment.

More rigid respiratory system designs (due to temporal expansion of the balloon’s volume, Table 6) could further reduce the registration and prediction errors. Further preliminary clinical trial with patients is planned and under way; due to the complexity of the trials, it is foreseen to be published separately.
